# Visualizing the heterogeneous breakdown of a fractal microstructure during compaction by neutron dark-field imaging

**DOI:** 10.1038/s41598-018-35845-y

**Published:** 2018-12-14

**Authors:** R. P. Harti, J. Valsecchi, P. Trtik, D. Mannes, C. Carminati, M. Strobl, J. Plomp, C. P. Duif, C. Grünzweig

**Affiliations:** 1 Laboratory for Neutron Scattering and Imaging, Paul Scherrer Institut, Zurich, Switzerland; 2 Niels Bohr Institute, Copenhagen, Denmark; 3 Reactor Institute Delft, TU Delft, Delft, Netherlands

## Abstract

Structural properties of cohesive powders are dominated by their microstructural composition. Powders with a fractal microstructure show particularly interesting properties during compaction where a microstructural transition and a fractal breakdown happen before compaction and force transport. The study of this phenomenon has been challenging due to its long-range effect and the subsequent necessity to characterize these microstructural changes on a macroscopic scale. For the detailed investigation of the complex nature of powder compaction for various densification states along with the heterogeneous breakdown of the fractal microstructure we applied neutron dark-field imaging in combination with a variety of supporting techniques with various spatial resolutions, field-of-views and information depths. We used scanning electron microscopy to image the surface microstructure in a small field-of-view and X-ray tomography to image density variations in 3D with lower spatial resolution. Non-local spin-echo small-angle neutron scattering results are used to evaluate fitting models later used as input parameters for the neutron dark-field imaging data analysis. Finally, neutron dark-field imaging results in combination with supporting measurements using scanning electron microscopy, X-ray tomography and spin-echo small angle scattering allowed us to comprehensively study the heterogeneous transition from a fractal to a homogeneous microstructure of a cohesive powder in a quantitative manner.

## Introduction

Powders form the foundation of many technological and fundamental achievements in fields such as the production of nanocrystals^1^ as well as the understanding of the complex interplay of forces^2–4^. The cohesiveness of powders is determined by the size of the individual particles and their grain morphology. If the particles are small enough forces that drive cohesion become dominant. Electrostatic, capillary and van der Waals forces impact the behaviour of cohesive powders and especially their compaction behaviour. Powder compaction makes it possible to understand the impact and strength of the different forces and is an important technological process when it comes to the production of nanocrystals. Besides the forces acting within the powder its microstructure is another important factor determining compaction behaviour. As pointed out in^5^ fractal powders are a special type of powder, with a compaction behaviour that differs compared to ballistically deposited powders. Fractal powders typically retain their structure until a critical force is applied that breaks down the fractal structure after which the powder^4^ behaves as if ballistically deposited. Despite the significance of the powder microstructure its characterisation and investigation has traditionally been a challenge due to the variety of length scales that are subject to change during powder compaction. Ranging from micrometre sized structures to macroscopic variations.

Typically, grain morphology of powders are studied using scattering methods with X-rays such as X-ray diffraction^6^ or small angle X-ray scattering^7^ and neutrons, such as spin-echo small-angle neutron scattering (SESANS)^8^. These methods however fail to deliver sufficient spatial resolution. High resolution X-ray tomography^9–11^ and scanning electron microscopy (SEM)^12^ are another approach for the study of microstructures that does give spatial resolution but fails to provide a relevant large field-of-view that is required to study heterogeneous behaviour on a macroscopic scale.

In this article we will use the neutron dark-field image (DFI) contrast^13,14^ of the neutron grating interferometry (nGI) method^15^ and especially the recently developed sub-pixel correlation length imaging (ξDFI) technique^16–18^ in combination with supporting methods such as SEM, X-ray computed tomography (X-CT) and SESANS to study the compaction of a cohesive powder. We investigate how compaction with a pyramidal shape, at various densification states, influences the microstructure over the range of centimetres and how it produces a heterogeneous breakdown of the fractal microstructure. Furthermore, we are able to visualize how the structural change from fractal to ballistic is transported through the macroscopic sample by utilising the unique capabilities of ξDFI to study microstructural variations in a large centimetre sized field-of-view that would not be accessible otherwise.

## Sample, Supporting Methods and Experimental Results

### Sample

The powder sample studied in this paper is the commercially available Sipernat-310. Sipernat is a series of products of precipitated silica, aluminium and calcium silicates^19^. Sipernat-310 is a silica powder designed to have a large surface area of 700 m^2^/g. The combination of large surface area with the relatively small particle size of 8.5 μm (d50) makes Sipernat-310 an ideal candidate to study cohesive powders, as these are the factors driving cohesion. In addition to the structural properties of Sipernat-310 the material composition of silica, SiO_2_, makes it a material with relatively low neutron absorption. The absorption cross-section for 4.1 Å neutrons is as low as 0.004 cm^−1^ ^20^. The low neutron absorption allows us to record dark-field data without many neutrons being attenuated and thus not contributing to the uncertainty of the dark-field signal^21^.

### Supporting methods

We start the analysis of the powder compaction with high-resolution SEM imaging to directly image the microstructure of the powder and verify its fractal as well as cohesive properties. However only small field-of-views can be realised and no *in-situ* powder compaction can be achieved. Furthermore, SEM is a surface sensitive technique, not giving information about the bulk properties of the sample. Therefore, we extend the direct imaging analysis by using X-CT to study agglomerates and larger structures. While X-CT offers a relatively large field-of-view and information about the bulk it does not enable the study of microstructural changes directly due to limited spatial resolution. Here the use of SESANS enables us to observe microstructural changes in the powder during compaction in the bulk, but without spatial resolution. The scattering nature of the technique makes it possible to verify scattering models that describe microstructural changes. All the knowledge gained from the supporting methods of SEM, X-CT and SESANS allowed us to use DFI and especially ξDFI to study the compaction of the powder sample with a pyramidal piston that introduces spatial variation in the microstructure on cm length scales.

### SEM results

A loose, not compacted, sample was prepared by pouring the Sipernat-310 powder on a sticky graphite tape, which was then mounted in the SEM. A compressed sample was prepared by using a mechanical device (cf. Fig. 6 in Method section) specially manufactured for the heterogeneous compaction of powders applying pressures in the range of ~100 kPa, which is later on also used in the ξDFI experiments. Once the powder was compressed the device was opened and the compacted powder has formed a stable structure which could then be broken off in fairly large, compressed pieces. These pieces were then glued to a graphite tape sample holder. In order to avoid charging effects the samples were coated with a thin graphite layer.

**Figure 1:**
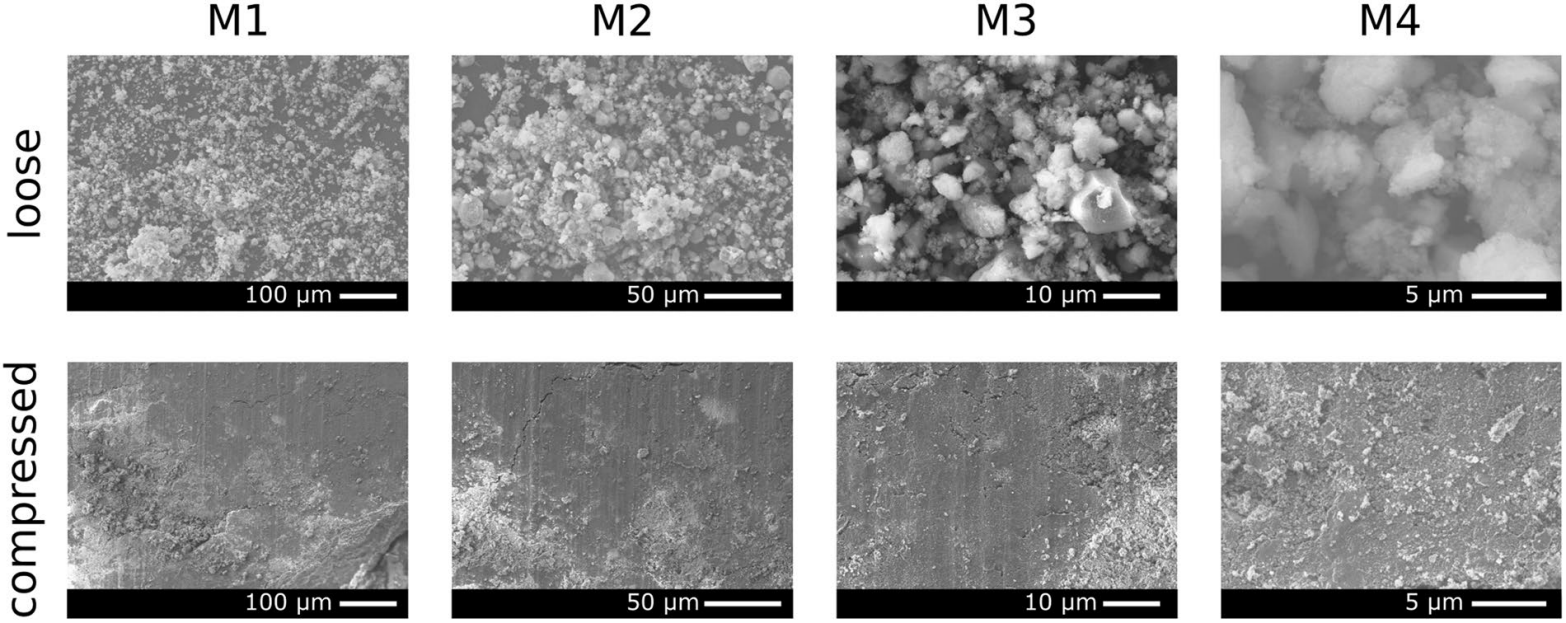
SEM images of a fractal cohesive powder (Sipernat-310) in a loose (top row) and compressed (bottom) state. The increase in magnification from M1 to M4 illustrates the random and fractal ordering of the loose packing, while an increase in magnification for the compacted sample does not show any significant changes, indicating a much less pronounced microstructure.

The first characterisation of the fractal powder by SEM was done by recording two sets of images as presented in Fig. 1. One of the loosely poured sample, top part of Fig. 1, for a variety of different magnifications (M1–M4) and one for a completely compressed sample, bottom part of Fig. 1. These two states represent the initial and final state of the sample throughout a compaction process that we will use to study the fractal breakdown as a function of compression throughout this work.

Initially the images of the loosely poured sample indicate a fractal microstructure, comparable to the sample presented in^5^. The presence of a variety of length scales in all magnifications confirms the initial assumption of a fractal structure of the loosely poured powder.

In addition to the confirmation of the fractal initial state of the powder the SEM data also supports the description as a cohesive powder, brought forward in^8^. Looking at the images of the compressed powder in Fig. 1 it becomes apparent, that a compaction of the powder leads to a very homogeneous material that exhibits virtually a more homogenous morphology that can be resolved with the SEM. This indicates, that the individual particles deform and stick together, justifying the characterisation of the sample as a cohesive powder with a fractal structure in its uncompressed state.

The high-resolution images obtained by SEM allow us to study individual particles of the powder in the loose and compressed state. The advantages of high spatial resolution measurements, such as SEM, are often set off by the small field-of-view. However local changes of microstructure can well be studied directly using SEM in a surface sensitive manner. In the following we continue with a bulk sensitive technique, X-CT.

### X-CT results

**Figure 2:**
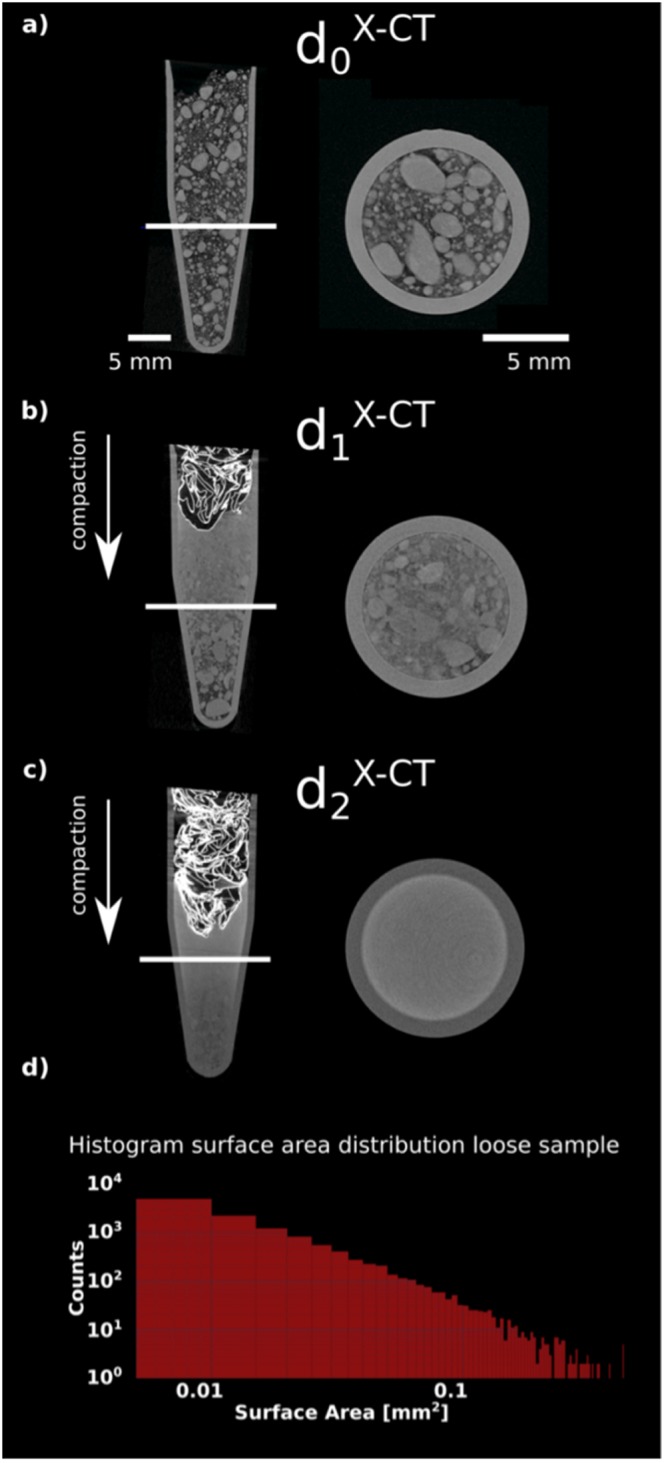
X-CT of a loosely poured for different densification states: (**a**) d_0_^X-CT^ (**b**) an intermediately compacted d_1_^X-CT^ and fully compacted d_2_^X-CT^ (**c**) Sipernat-310 powder. The brighter structure on top of the powder in (**b**,**d**) is aluminum foil used for compaction. The right images are a cut through the sample at the location indicated by the white lines. An increase in compaction shows a stronger homogeneity, similar to the SEM measurements. The histogram (**d**) illustrates the distribution of agglomerate sizes in the poured sample. Its relatively straight shape indicates fractal behaviour.

The initial fractal composition of the powder also manifests itself in an inhomogeneous density distribution for larger length scales^5^. We probed this characteristic using X-ray tomography. The results of three different densification states *d*^*X*−*CT*^ are shown in Fig. 2.

The sample holder was a cylindrical plastic tube that has a conical shape on the bottom, typically used for centrifuge applications. The powder was compressed using a plug made of aluminium foil. In this sample container we prepared the Sipernat-310 powder in three different densification states: the poured sample without any compaction *d*_0_^*X*−*CT*^, an intermediate compaction state *d*_1_^*X*−*CT*^ and a compacted state *d*_2_^*X*−*CT*^.

The poured sample shows large inhomogeneities, that well extend the micrometre sized particles as probed by the SEM. This can be observed in the tomographic slices in Fig. 2a. These inhomogeneities are an indication for the formation of agglomerates in which the individual micro particles cluster together due to cohesion^22^. We used the tomography of the poured powder to study the characteristics of the agglomerates by determining the surface area of the agglomerates and a histogram plot is shown in Fig. 2d. We have chosen to investigate the surface area of the agglomerates as it is an important parameter impacting both the cohesiveness and the fractal properties of the powder. As pointed out in^23^ a linear behaviour in the loglog plot indicates a fractal distribution, conforming a fractal structure also for the relatively large structures probed by X-ray tomography.

The tomography of the intermediate compaction state illustrates the behaviour the agglomerates exhibit during compaction as shown in Fig. 2b. Especially at the edges and on top of the sample (the areas with largest pressure during compaction) we observe a gradual disappearance of the inhomogeneities. The non-uniform behaviour over the whole sample indicates that the sample is first homogenised, before transporting the pressure of compaction to lower layers.

At the most compacted state of the sample there are almost no inhomogeneities observable anymore as seen in Fig. 2c. This means all agglomerates were broken and no fractal behaviour can be observed anymore in the sample. It became, on the probed length scale, homogeneous. This behaviour is identical to the observations in the SEM measurements, but on a length scale several orders of magnitude larger. This indicates a comparable behaviour of the sample in the micrometre as well as millimetre range.

The combined information of SEM and X-CT allow us to draw the conclusion, that the cohesive powder exhibits a change from a fractal to a homogeneous structure for all size ranges accessible with ξDFI^17^. The SEM results showed the behaviour for the microstructure and X-CT data illustrated the macroscopic behaviour with an imaging resolution similar to the spatial resolution in ξDFI. In the next section we will verify a scattering model that describes the microstructure of the sample, which will then later be used to fit the ξDFI data. As SESANS gives the same scattering functions as each pixel in ξDFI we conducted a SESANS experiment of the powder compaction.

### SESANS results

The verification of the fractal nature of the poured sample and its breakdown during compaction lead us to record SESANS data in order to quantitatively characterise the microstructure. This way we are able to extract parameters, which characterise the microstructure and observe changes during the fractal breakdown however on a non-local or global probing volume.

**Figure 3:**
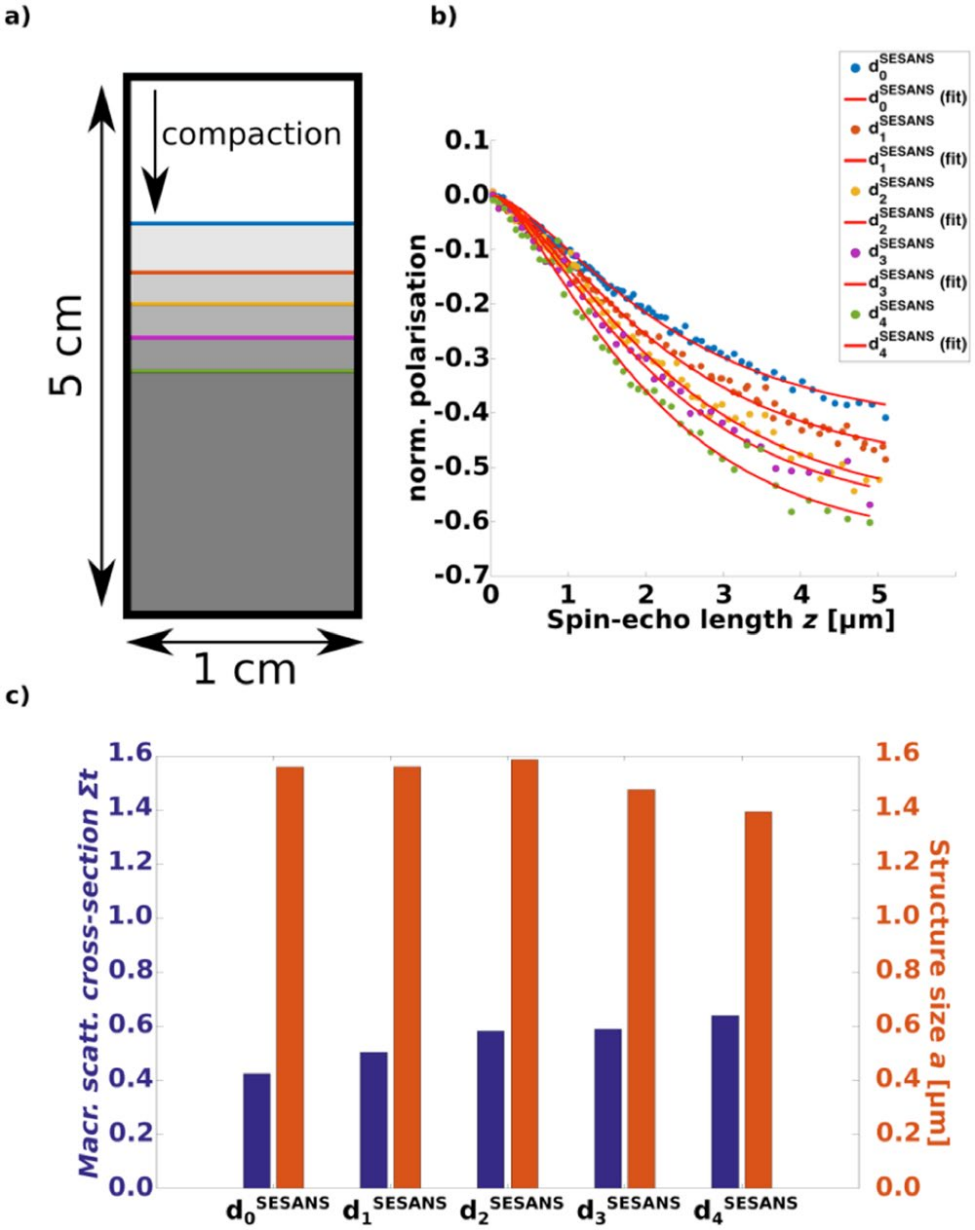
SESANS study of powder compaction. (**a**) Schematic illustration of the compaction steps within the sample container during the experiment. The blue line resembles d_0_^SESANS^, the red line d_1_^SESANS^, the yellow line d_2_^SESANS^, the purple line d_4_^SESANS^ and the green line d_4_^SESANS^ (**b**) Evolution of SESANS signal with increased compaction of the sample. SESANS curve and fit at each densification. (**c**) Fitting parameters giving macroscopic scattering cross-section and inhomogeneity size. Extracted from the fits in (**b**).

For the SESANS experiments we used a cuvette of non-borated quartz glass with a thickness of 5 mm as shown in Fig. 3a. The compaction was performed using a specifically manufactured aluminium piece as a plug.

SESANS data is typically plotted as spin-echo length versus polarisation as shown in Fig. 3b, where spin echo length *z* represents the probed structure size in the sample. The data is described by^8^

$$P(z)={e}^{\Sigma t(G(z)-1)}$$

where *P* is the normalised polarisation of the neutron beam after passing the sample, *Σ* is the macroscopic scattering cross-section, *t* the sample thickness and G the projected density correlation function^24^. *Σ* is an indication for the probability of a neutron scattering with the sample while transmitting it and is defined by^8^ as

$$\Sigma ={\lambda }^{2}\Delta {\rho }^{2}\Phi (1-\Phi )\chi $$

Here *λ* is the probing wavelength, *Δρ* the difference in coherent scattering length density of the scattering contributors, *Φ* the volume fraction of the scatterer and *χ* the characteristically probed structure size of the sample.

While *Σ* purely gives information about the total probed sample interaction *G* reveals information about the internal microstructure of the sample which determines the shape of a SESANS curve. According to^8^ randomly distributed two phase media, such as the powder studied in this work, can be modelled by

$$G(z)=\frac{z}{a}{K}_{1}(\frac{z}{a})$$

with *a* being the characteristic structure size of the powder and *K*_1_ the modified Bessel function of second kind and first order.

As defined in^8^ the autocorrelation function representation *γ(r)* of *G(z)* is

$$(r)={e}^{-\frac{r}{a}}.$$

*χ* is defined as

$$\chi =2\,{\int }_{0}^{\infty }\gamma (r)dr$$

The combination of Equations for γ(r) and *χ* leads to the relation of *χ* and a for the model used here of

$$\chi =2a.$$

We used this model to fit the SESANS measurements for five different densification states, from loosely poured sample in d_0_^SESANS^ to strongly compacted d_4_^SESANS^, of the powder as seen in Fig. 3a). The sample is a cuvette initially filled up to the top blue line resembling d_0_^SESANS^. The sample is compacted stepwise with the indicated depths all the way to the green line on the bottom, resembling d_4_^SESANS^. Figure 3b presents the extracted SESANS functions and the fits of the model of *G(z)* for each compaction. We recorded the extracted parameters in Fig. 3c). One can see that the fits in Fig. 3b) well describe the data and we are thus confident to have chosen the right model, which we will use again later for measurements unifying the spatial resolution of imaging with the microstructure characterisation of the projected density correlation function.

During powder compaction the extracted *Σt* and characteristic structure sizes *a* from the SESANS curves indicate behaviours that allow the observation of fractal breakdown of the microstructure as an averaged signal over the whole sample, thus representing a mixture of fractal and non-fractal structure. From densification state d_0_^SESANS^ − d_2_^SESANS^ in Fig. 3c the characteristic length of the microstructure stays constant while *Σt* increases. This indicates an increase in concentration without introducing structural changes, keeping the fractal microstructure intact.

Moving from d_2_^SESANS^ to d_3_^SESANS^ the characteristic structure size decreases while *Σt* stays constant, indicating a breakdown of the fractal microstructure, which is accompanied by a change in characteristic structure size. The constant *Σt* is a consequence of an increased concentration, which is offset by the change in structure size. The combined behaviour of decreased characteristic structure size with constant *Σt* thus indicates a phase transition from fractal to ballistic microstructure in progress.

Densification d_4_^SESANS^ to d_5_^SESANS^ is characterised by a decrease in characteristic structure size while at the same time an increasing *Σt*. This is explained by a further compaction of the ballistic structure without additional phase transitions to other microstructures. Thus, the random orientation of a ballistic system is further compressed and inhomogeneities have disappeared, as seen in the X-ray tomographies and the SEM images of the compacted powder.

### Heterogeneous powder compaction studied with ξDFI

The combination of SEM, X-CT and SESANS illustrates the behaviour of the powder during compaction for multiple length scales and made it possible to find a scattering model that describes the microstructure of the powder. In the following we apply ξDFI to study this behaviour during compaction with a pyramidal shaped piston. We refer to ξDFI as the method of changing the autocorrelation length ξ of the neutron grating interferometer, which is defined by^16^

$$\xi =\frac{\lambda {L}_{s}}{{p}_{2}}$$

Here *λ* represents the incoming wavelength of the neutron, *L*_*s*_ the sample to G2 grating distance and *p*_2_ the period of G2 (cf. Fig. 6 in Experimental Section). By variation of *ξ* we observe a change in contrast in the DFI signal, that is related to the probed microstructure. The contrast variation with varying *ξ* allows us to deduct the projected density correlation function *G(ξ*) of the sample in each pixel of an image. Thus, we are able to produce spatially resolved function of the sample that characterises the microstructure of the sample pixelwise with scattering functions, that are the same as measured in SESANS^16^.

**Figure 4:**
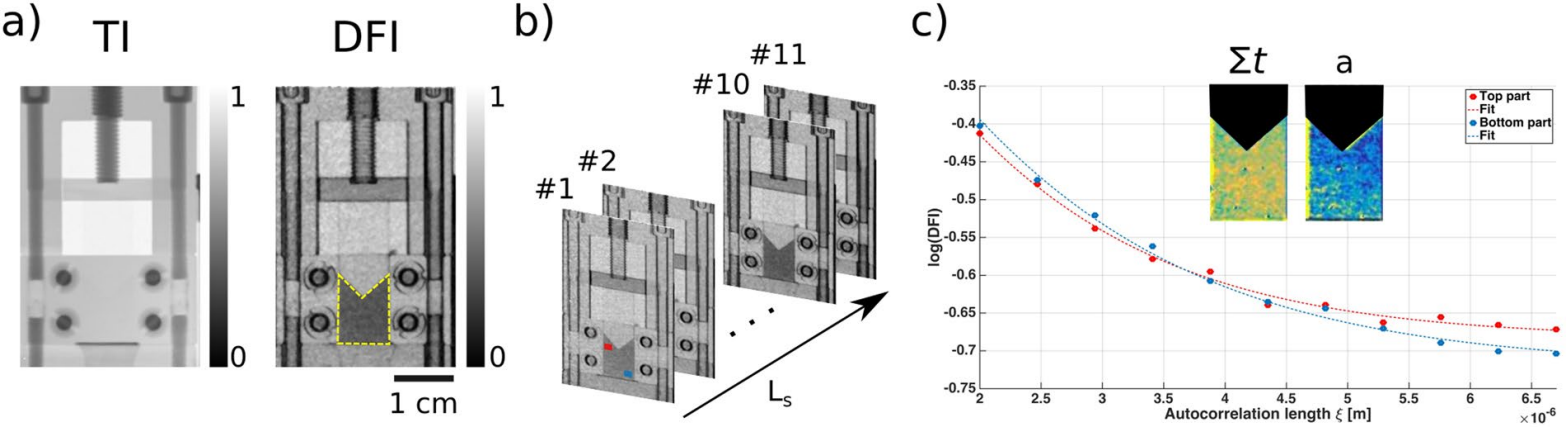
(**a**) Transmission image (TI) and dark-field image (DFI) of the compaction device and the volume filled with the powder (yellow dashed line). (**b**) Data acquisition procedure for ξDFI data generation. 11 DFIs are recorded at equidistant L_S_ values. (**c**) Plot of the DFI data vs ξ for two different areas as marked with the red and blue points in (**b**) and the corresponding fits. From the fit values the parameter maps of the macroscopic scattering cross section Σt and characteristic structure size a are extracted.

The compaction device as shown in Fig. 4a) with a pyramidal shaped piston induces inhomogeneous force distributions throughout large parts of the powder. The combination of scattering function in each pixel and the macroscopic imaging resolution of ξDFI enabled us to study the effect of the inhomogeneous force distribution on the breakdown of the fractal microstructure. With ξDFI we are able to combine microstructural characterisation with spatial resolution of imaging. This way we are able to investigate the heterogeneous breakdown of the fractal microstructure while compressing the powder.

In order to extract the projected density correlation function, we recorded DFI data sets at 11 equidistant values of L_s_ as shown in Fig. 4b) and thus varied the autocorrelation length of the setup from 2 to 7 μm. For each distance a full set of transmission and dark-field image was extracted using the open-access in-house software *TaPy*^25^. The change in dark-field signal for the variation in distance was analysed with a MATLAB script that fits the change in each pixel with a custom function as shown in Fig. 4c). In order to increase the statistics in each pixel a 4 × 4 binning on the reduced data was performed before fitting.

The recording of DFIs at varying sample to G2 distances allows us to scan the autocorrelation length of an imaging setup by tuning the parameter*ξ*. This experimental approach allows us to scan the same functions as in SESANS measurement, but in each pixel of an image, by varying *ξ*, which is analogous to the spin echo length *z*. Thus, we a are able to extract the fitting parameters presented in Fig. 3c) spatially resolved and *in-situ*. This is possible due to the high penetration power of neutrons.

The DFI contrast in neutron grating interferometers is described by the same equation as SESANS:

$$DFI(\xi )={e}^{\Sigma t(G(\xi )-1)}$$

Thus, we use the same fitting model that has proven to describe the microstructure well in the SESANS measurements to extract *Σt* and the characteristic structure size *a* as shown in Fig. 4c of the sample spatially resolved.

We built a powder compaction setup from Aluminium, as illustrated by the sample in Experimental section Fig. 6, in which we placed a 1 mm layer of the Sipernat-310 sample. This 1 mm layer is then impinged and compacted by a pyramidal shaped aluminium piston to introduce an uneven force distribution during compaction. We recorded data at no compaction (d_0_^DFI^), two intermediate compaction states (d_1_^DFI^ and d_2_^DFI^) and one fully compacted state (d_3_^DFI^). At each of the compaction states we recorded a full scattering function in each pixel of the image and fitted the model and created maps of the fitting parameters *Σt* and *a*.

**Figure 5:**
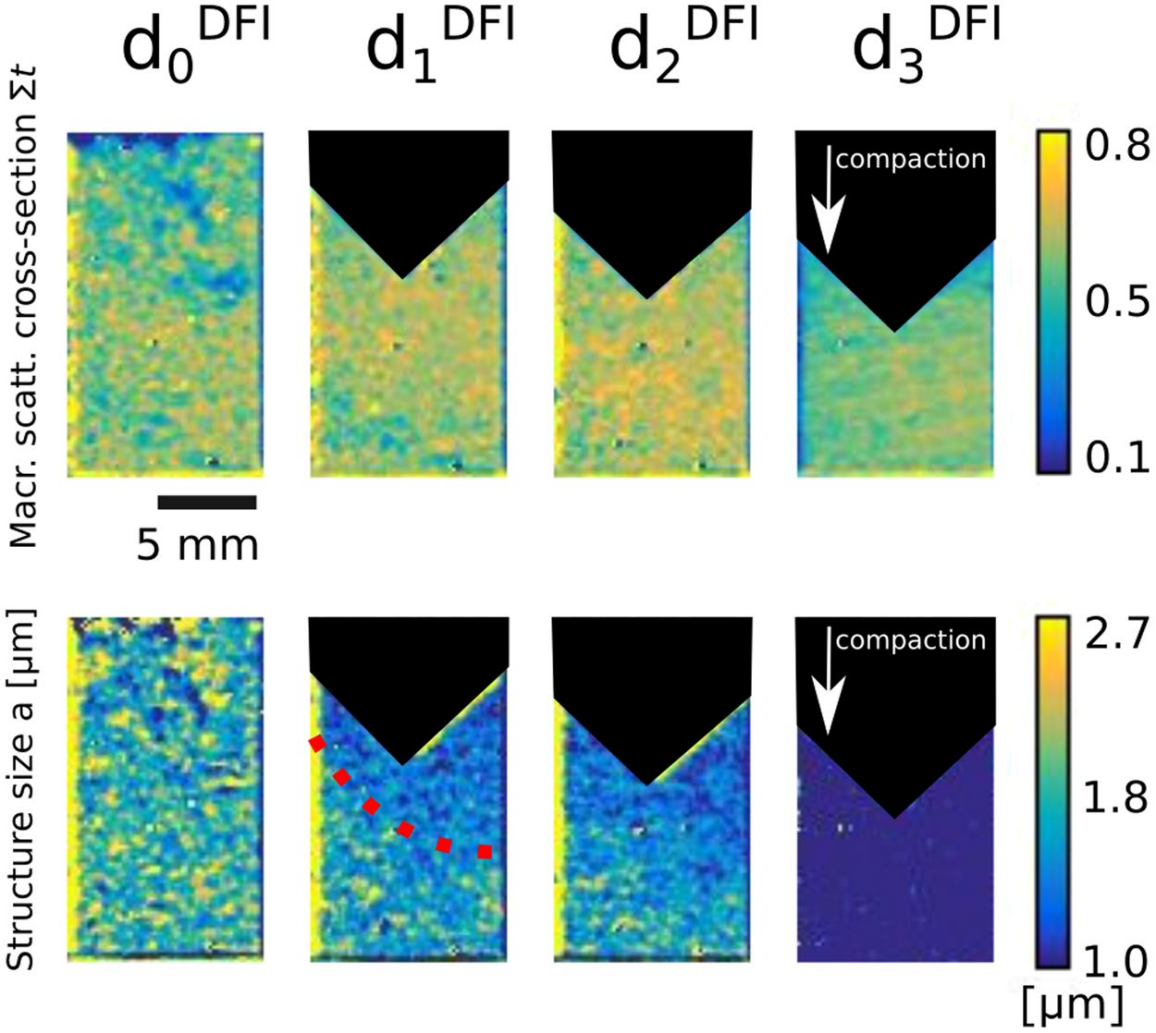
Quantitative ξDFI results: Maps of the macroscopic scattering cross-section Σt and characteristic structure size a of 4 different densification states d^DFI^ of the compaction of a 1 mm layer of Sipernat-310 powder densified with a pyramidal shaped aluminium piston. The maps were extracted from fitting the model for random two-phase media to neutron dark-field images with variation of the autocorrelation length ξ of the imaging setup. The dotted red line indicates the propagation front of the structure breakdown.

Figure 5 shows the results for compaction d_0_^DFI^ to d_3_^DFI^. The top part of the Figure presents the *Σt* maps of the powder at varying compaction states. The values correspond to the blue bars in Fig. 3c) with the additional benefit of spatial resolution. The densification does not impact *Σt* significantly and only visualises the change from a macroscopically heterogeneous system to a homogeneous system.

The parameter maps in the bottom part of Fig. 5 present a change of the microstructure and the values correspond to the red bars in Fig. 3c.) As shown before a change in value of this parameter indicates a change in microstructure from fractal to ballistic. This can be observed at densification states d_1_^DFI^ and d_2_^DFI^ where close to the impinging pyramid, especially in the corners, the fitted values show a significantly decrease in comparison to the not compacted state in d_0_^DFI^. The smaller value extends downward with a propagation front that becomes larger for d_2_^DFI^ compared to d_1_^DFI^. The bottom of the sample does not show any change in microstructure in d_1_^DFI^ and d_2_^DFI^ and stays the same as in d_1_^DFI^. Thus, we observe the breakdown of the fractal microstructure of the powder at the edges of the pyramid and extended towards the bottom of the sample where the fractal nature is still intact.

The final compaction state d_3_^DFI^ corresponds to the bottom part of Fig. 1 and does not show significant microstructure anymore. This is confirmed by the very strong decrease of a in d_3_^DFI^. This state exhibits no heterogeneities anymore for all probed length scales, from SEM images over X-ray tomographies to the DFI measurements.

### Heterogeneous fractal breakdown

The combined information from SEM, X-CT, SESANS and ξDFI illustrates the structural change of a cohesive powder from a fractal microstructure to a homogeneously compacted state. As pointed out in^5^ this behaviour is an important factor for compaction properties of a powder. We observed the homogenising behaviour for length scales of μm in SEM to mm in X-ray tomography.

The phase transition from a fractal structure of the loosely poured powder to a homogenous distribution is characterised by a decrease in size of the inhomogeneities measured using SESANS. This breakdown of the fractal structure does not happen immediately but only after some initial compaction, as proposed in^5^. We used the SESANS results to verify scattering models by fitting them to the SESANS data. We then used this model for the DFI data to extract spatially resolved information.

The parameter maps in Fig. 5 illustrate the behaviour of the powder under compaction for a pyramidal piston impinging the powder. The loose powder shows density inhomogeneities but is not characterised by a heterogeneous microstructure. After an initial compaction we observe a general increase in density, as expected. The characteristic size of the inhomogeneities however shows strong heterogeneous behaviour.

Just under the impinging pyramid we observe a change in parameter *a*. This is the result of forces acting upon the powder and causing the fractal structure to collapse. This also shows that while some of the force is transported through the powder, as evident by the partial homogenisation of the density parameter *Σt* it is not enough to break the fractal in the bottom of the sample. The fractal breakdown rather happens as a propagation front and covers an increased part of the sample with increased compaction.

At full compaction the samples microstructure is homogeneous, in accordance with the SEM measurements and does not show any inhomogeneities anymore. Thus, we observe the breakdown of the fractal structure in parts of the sample during compaction, while other parts still retain their fractal structure.

This measurement confirms the contact dynamics simulation (CDS) as reported in literature^5^, in Figure 12 in the reference. The simulation suggests that a compaction of an initially loose, cohesive powder leads to a fractal breakdown close to the piston coming from the top. The subsequent breakdown of the fractal structure in the whole sample, then happens by pressure transport through a propagation front. This is in accordance with the ξDFI measurements in Fig. 5 where the measured *a* initially drops close to the pyramidal piston. This is an indication for the breakdown of the fractal microstructure, as argued above. The measurements show how ξDFI offers unique insight into the fractal breakdown of a powder structure and shows that the behaviour is in good agreement with simulations that show a behaviour that has not been visualised in transmission experimentation before.

## Conclusion

The experimental data presented in this work shows the application of a set of characterisation techniques to comprehensively study the heterogeneous transition from a fractal to a homogeneous microstructure. We combined SEM imaging with high resolution with X-CT with a large field-of-view to investigate the compaction of a cohesive powder at the micro and macro scale. In order to comprehensively study the microstructural changes we applied SESANS and confirmed a scattering model that describes two-phase random media. During compaction we were able to present the evolution of the characteristic size of the inhomogeneities in the sample.

The combined knowledge gained from SEM, X-CT and SESANS was then used to apply ξDFI to a compaction experiment of the same powder using a pyramidal shaped piston that introduces spatial variations in the sample. The combination of macroscopic imaging resolution with microstructural information by fitting the model confirmed with SESANS enabled us to visualise the propagation front of the breakdown of the fractal microstructure using ξDFI.

## Methods

### SEM

The scanning electron microscope used for the results presented in this work was a ZEISS NVISION microscope. Eight images have been recorded at four magnifications (M1–M4) of 200x, 500x, 2000x and 5000x with corresponding pixel sizes of 560 nm, 223 nm, 56 nm and 22 nm, respectively.

### X-ray tomography

The X-ray tomography experiment was conducted at the ICON beamline at the Paul Scherrer Institut using its X-ray tomography option^26^. The X-ray source is a Hamamatsu L212161-07 micro focus tube which was operated at 60 kV tube voltage and 100 μA tube current. A Varian 2530HE flat panel detector was used. The detector was configured to collect 16-bit images with a gain setting of 2 and 5 seconds exposure time per sample rotation position. A total of 626 projections have been recorded over 360 degrees adding up to 52 minutes exposure time per tomography.

### Spin-echo small-angle neutron scattering

The SESANS data was recorded at HOR Reactor at the Reactor Institute at TU Delft, Netherlands. Spin Echo SANS is performed at a dedicated beamline that can probe structure sizes from 20 nm up to 20 μm using a monochromatic beam of 2 Å.

### Neutron grating interferometry

**Figure 6:**
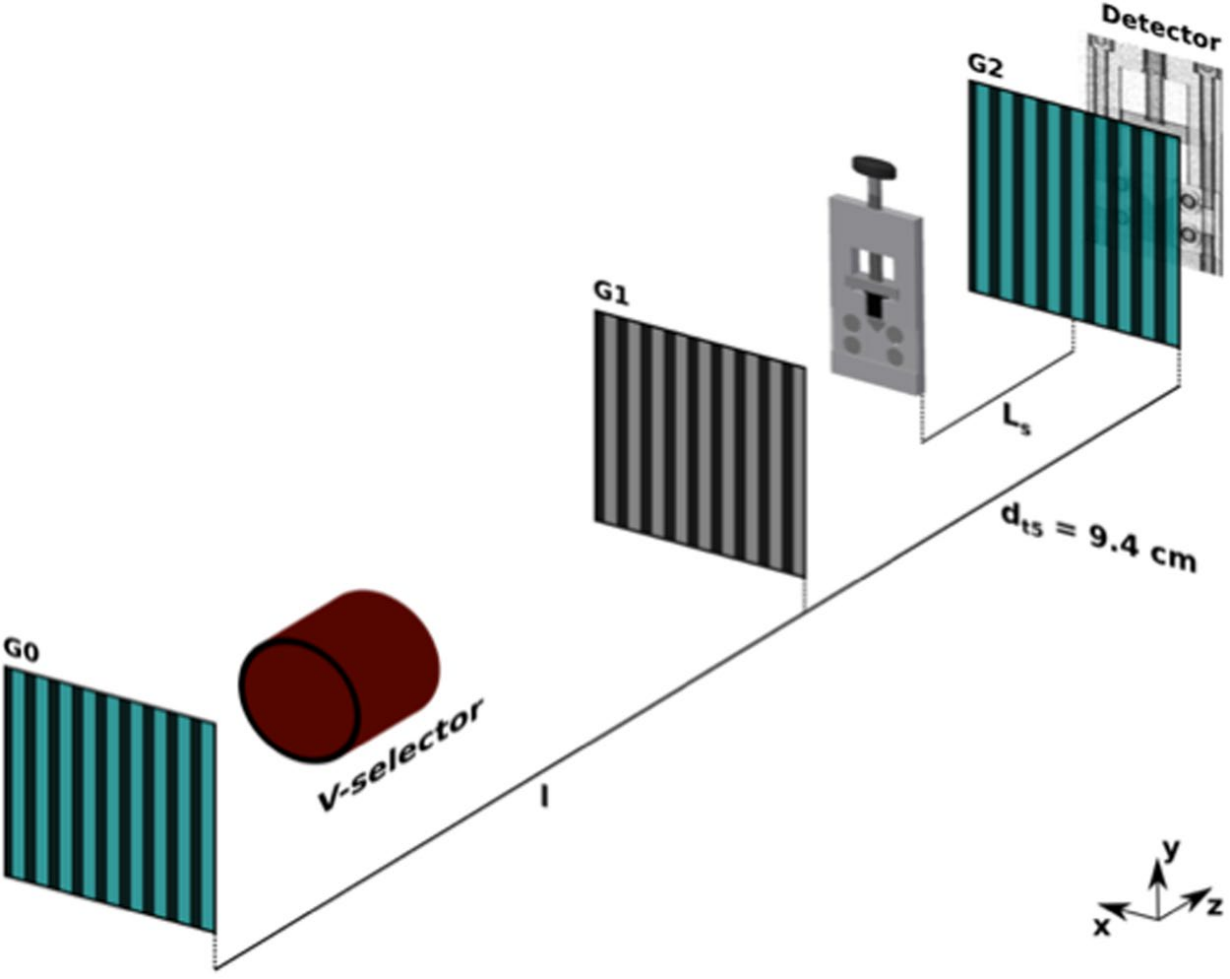
Sketch of the nGI setup for the fifth Talbot distance. G0 to G1 distance is 5.23 m and the G1 to G2 distance is 9.4 cm resembling the fifth Talbot distance. The sample is positioned between G1 and G2 and is represented by the powder compaction device described used for the ξDFI as well as the SEM experiment. The detector shows an example DFI of the powder in the compaction device.

The ξDFI experiments were conducted at the beamline ICON at PSI. A dedicated setup at the 5th Talbot distance was used. A sketch of the setup is shown in Fig. 6 and the detailed setup parameters, as well as the accessible ξ range of the nGI setup are presented in Table 1. The neutron grating interferometer data consists of multiple images that discreetly record the stepping of one of the gratings over a full period. In our case we stepped G0 with 19 steps over 222 μm and recorded images at each step with an exposure time of 105 sec.

**Table 1:** Setup details for the nGI used during the investigation of powder compaction.

d_tn_	p_0_ [μm]	p_1_ [μm]	p_2_ [μm]	l [m]	d [cm]	λ [Å]	ξ scan parameter	ξ range [μm]
5	222	7.86	4	5.23	9.4	4.1	L_s_	2–7

d_tn_: Talbot order; p_0_: Period G0; p_1_: Period G1; p_2_: Period G2; l: Distance G0–G1; d: Distance G1–G2.
